# The Hypoxic Response Expression as a Survival Biomarkers in Treatment-Naive Advanced Breast Cancer

**DOI:** 10.31557/APJCP.2020.21.3.629

**Published:** 2020-03

**Authors:** Irwan Gunawan, Mochammad Hatta, Andi Fachruddin Benyamin, Andi Asadul Islam

**Affiliations:** 1 *Department of General Surgery, *; 2 *Biology Molecular and Immunology Laboratory, *; 3 *Division of Medical Oncology-Hematology, *; 4 *4Department of Surgery, Faculty of Medicine, Hasanuddin University, Makassar, Indonesia.*

**Keywords:** Breast neoplasms, carbonic anhydrase IX, hypoxia-Inducible Factor 1, tumor hypoxia, survival

## Abstract

**Objective:**

Hypoxia-associated biomarkers profiling may provide information for prognosis, staging, and subsequent therapy. We aim to evaluate whether the quantitative gene and protein expression of hypoxic response tumor markers — carbonic anhydrase IX (CAIX) and hypoxia- inducible factor 1 alpha (HIF1A) — may have a role in predicting survival in advanced breast cancer of Indonesian population.

**Methods:**

Tumor tissues and peripheral blood samples were collected from treatment - naïve locally advanced (LABC) or metastatic breast cancer patients (MBC) at Wahidin Sudirohusodo General Hospital (Makassar, South Sulawesi) and its referral network hospitals from July 2017 to March 2019. The level of mRNA (of blood and tumor tissue samples) and soluble protein (of blood samples) of CAIX and HIF1A were measured by RT-qPCR and ELISA methods, respectively, besides the standard histopathological grading and molecular subtype assessment. The CAIX and HIF1A expression, patients’ age, tumor characteristics, surgery status, and neoadjuvant chemotherapy drug classes were further involved in survival analyses for overall survival (OS) and progression-free survival (PFS).

**Results:**

Forty (30 LABC, 10 MBC) eligible patients examined were 21 hormone-receptors positives (15 Luminal A, 6 Luminal B) and 19 hormone-receptors negatives (10 HER2-enriched, 9 triple-negative). The CAIX blood mRNA and CAIX soluble protein levels in hormone-receptors negative patients were higher than in hormone-receptor-positive patients (p < 0.05). In univariate analysis, both CAIX and HIF1A levels predict OS (except HIF1A protein) with CAIX tissue mRNA has the highest hazard ratio (HR 8.04, 95%CI:2.45-26.39), but not PFS. Cox proportional hazard model confirmed that CAIX tissue mRNA is the independent predictor of OS (HR 6.10, 95%CI: 1.16-32.13) along with surgical status and tumor advancement type (LABC or MBC).

**Conclusions:**

CAIX mRNA expression of tumor tissue in treatment-naïve advanced breast cancer has a predictive value for OS.

## Introduction

Breast cancer is leading cancer in females worldwide (Ferlay et al., 2015). In the Asia-Pacific region, breast cancer incidence reached 18% of all cancers (Youlden et al., 2014). In South East Asian countries, the incidence and mortality are rising exceeds that of the developing countries (Bhikoo et al., 2011). In Indonesia, breast cancer remains a national health burden, due to early peak age at presentation with large, aggressive tumors and more advanced stages (Ng et al., 2011; Wahidin et al., 2012). Breast cancer is characterized by heterogeneous clinicopathologic features and a wide epidemiological spectrum. Thus, survival may vary regarding racial or ethnical differences (Warner et al., 2015). Improving characterization of an individual’s prognosis in advanced breast cancer in Indonesian women potentially aid their clinical management, e.g., by providing adequate information to help with planning and identifying patients with worst risk who may choose to participate in novel investigational therapies.

The current breast cancer treatment options are determined by the combined status of three key receptors; estrogen receptor (ER), progesterone receptor (PR) and human epidermal growth factor receptor 2 (HER2), resulting in four molecular subtypes (Luminal A, Luminal B, HER2-enriched, and triple negative) which differ in their gene expression patterns, clinical features, treatment response, and prognosis (Schnitt, 2010). Despite the application of molecular subtyping, identification of which cases will develop metastasis or relapse remains challenging. Expression of metabolism-related proteins seems to have different patterns regarding the molecular subtypes (Choi et al., 2013).

Intratumoral hypoxia has been identified as a predictor of metastasis independent of histopathology status (Semenza, 2012). Breast cancer cells respond to decreased oxygen availability by activating the hypoxia-inducible factors (HIF-1 and HIF-2), which regulate multiple genes involved in metastatic progression (Gilkes and Semenza, 2013). The carcinogenic role of HIF-1 alpha (HIF1A) may change from the response to proliferation to tumor progression (Chen et al., 2010). When hypoxic environment advances, HIF1A is overexpressed and promoting upregulation of various target genes, including carbonic anhydrase IX (CAIX), a transmembrane glycoprotein to prevent intracellular acidosis, allowing breast cancer cells to undergo metabolic adaptation to hypoxia (Chen et al., 2010; Choi et al., 2013). 

Recent meta-analyses showed that HIF1A (Wang et al., 2014) and CAIX (van Kuijk et al., 2016) overexpression are predictive of poor prognosis. However, all studies in those meta-analyses employed immunohistochemistry (IHC) to assess protein expression of HIF1A and CAIX, which is regarded as semi-quantitative method and subject to a certain degree of inter- and even intra-observer variability which makes scoring prone to discordance. Reverse-transcription quantitative real-time PCR (RT-qPCR) has been established as a qualitative and quantitative method that is rapid, accurate, and sensitive for comprehensive genomic, mRNA profiling (Murphy and Bustin, 2009). 

A methodological comparison between protein expression measurement of ER, PR, and HER2 by IHC versus their respective mRNA levels by RT-qPCR showed that detection of mRNA levels by RT-qPCR is a better approach for subtyping breast cancer and predicting the prognosis (Du et al., 2013; Wirtz et al., 2016). Enzyme-linked immunosorbent assay (ELISA) has also proven as a reliable, more objective quantitative method for measuring circulating CAIX protein in comparison to the standard IHC procedure (Liao and Lee, 2012).

In recent years, diagnostic and therapeutic agents targeting HIF1A and CAIX have been developed (Ward et al., 2013). Hypoxia-associated biomarkers profiling in advanced breast cancer may provide additional information for staging, clinical decision, prognosis, and potentially have a crucial part in the development of personalized therapeutic drugs. Using RT-qPCR and ELISA methods, we aim to evaluate whether the expression of mRNA in blood and tumor tissue and circulating protein of CAIX and HIF1A (along with established risk factors) have potentials to predict survival in treatment-naïve Indonesian with locally-advanced (LABC) and metastatic breast cancer (MBC).

## Materials and Methods

This study was conducted upon ethical clearance approval (no.: 53/H4.8.4.5.31/PP36-KOMETIK/2018) from the institutional review board at Universitas Hasanuddin, Makassar, South Sulawesi, Indonesia. Consecutive, treatment-naïve, and eligible LABC or MBC patients admitted to Wahidin Sudirohusodo General Hospital (Makassar, South Sulawesi) or its local referral network hospitals between July 2017 and March 2019 were recruited following written informed consent. Diagnosis of LABC or MBC was established by referring physicians, based on clinical examinations or medical imaging. Standard physical examination, routine laboratory workup, and medical imaging (x-ray, CT scan or ultrasonography) were later performed to confirm patient’s eligibility (lymph node involvements, far metastatic lesions) and to exclude concomitant malignancy. The inclusion criteria were: age >18 years old and diagnosed as LABC or MBC with histological type of invasive ductal carcinoma. Patients with histological type other than invasive ductal carcinoma or refused to participate in this study were excluded.

*Histopathology and immunohistochemistry*

Besides the tumor size measurement, standard histopathological evaluation, including histological type assessment and grading were performed following guidelines from the American Society of Clinical Oncology and the College of American Pathologists. Standard IHC method for ER, PR, and HER2 was utilized to further categorize tumor molecular subtype as hormone-receptors (HR)-positive (Luminal A or Luminal B) and HR-negative (HER2-enriched or triple-negative) (Schnitt, 2010).

*Hypoxia markers expression measurement*

Tumor tissue and peripheral blood obtained from each patient within their initial biopsy were subject to sample preparation for RT-qPCR analysis of CAIX and HIF1A mRNA (details in Suppl. 1). Additionally, a peripheral blood sample was also preserved for CAIX and HIF1A soluble protein measurement using ELISA kits according to the manufacturer’s protocols (details in Suppl. 2). 

*Statistical analysis*

Statistical analysis was performed using MedCalc for Windows version 18 (MedCalc Software, Ostend, Belgium, www.medcalc.com) and GraphPad Prism version 6.07 (GraphPad Software, La Jolla, California, USA, www.graphpad.com) with the signiﬁcance level was set at p <0.05. Patient characteristics, as well as the difference between expression levels of CAIX and HIF1A, were evaluated based on molecular subtypes. Correlation coefficients (r) were calculated between expression levels of CAIX and HIF1A and age and histopathological grades. The cut-offs of each CAIX and HIF1A parameter were decided based on the normality of data distribution (mean value is used when the data is normally distributed, while median if otherwise).

Survival analyses for overall survival (OS) and progression-free survival (PFS) were performed in both univariate and multivariate methods. An event for OS is defined as death by any cause, while for PFS as a breast cancer-related (or -suspected related) clinical progression (e.g., but not limited to the appearance of metastatic lesions, axillary lymph node swelling, and pleural effusion). Time unit was described as the number of days recorded from the day of blood and tissue sampling for initial biopsy.

Kaplan-Meier curves and log-rank test were used to analyze individual prognostic factors, while Cox proportional hazard models were built for simultaneously evaluate multiple prognostic factors. The variables involved in multivariate analysis were 1) protein and mRNA levels of CAIX and HIF1A, 2) age, 3) histopathological grades, 4) molecular subtypes (and HR status), 5) drug types for neoadjuvant chemotherapy (taxane-based, anthracycline-based, or hormone-based), 6) surgical status (underwent mastectomy or not), and 7) tumor advancement (LABC or MCBC). 

The assumptions for a model of Cox proportional hazard can be built (a linear relationship between each variable with the outcome; no multicollinearity among predictor variables) were checked with correlation analyses (limit r = 0.7). Variable selection method was backward, with inclusion level p < 0.157 and exclusion level p > 0.2. The prerequisite for the final model are 1) at least one parameter of either CAIX or HIF1A is included and significantly contributed to the model, and 2) the p-value of the overall model fit is statistically significant.

## Results

*Patient and tumor characteristics*

Forty (30 LABC, 10 MBC) eligible patients were aged between 28 to 83 years (mean 50.18 ± 12.05, median 49.50). They were 21 HR-positives (15 Luminal A, 6 Luminal B) and 19 HR-negatives (10 HER2-enriched, 9 triple-negative). Only one patient with histological grade I was observed. No significant difference in mean age, age group (< 40 y.o. vs. ≥ 40 y.o.), tumor type (LABC or MBC) and histological grade among HR presence or molecular subtype groups. Table 1 summarized the baseline characteristic of these patients. 

*CAIX and HIF1A expression*

**Figure 1:**
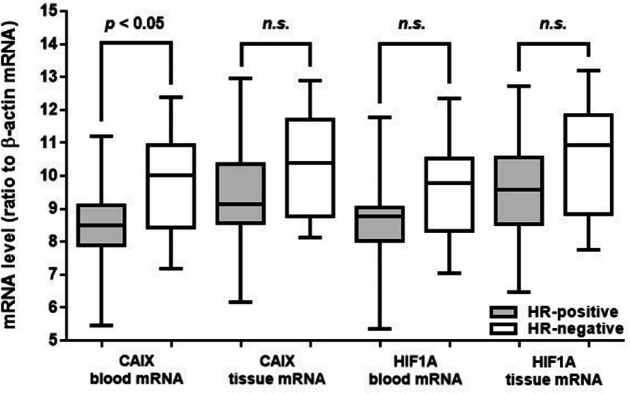
Distribution of mRNA Levels of CAIX and HIF1A from Blood and Tissue Samples based on HR Presence

**Figure 2:**
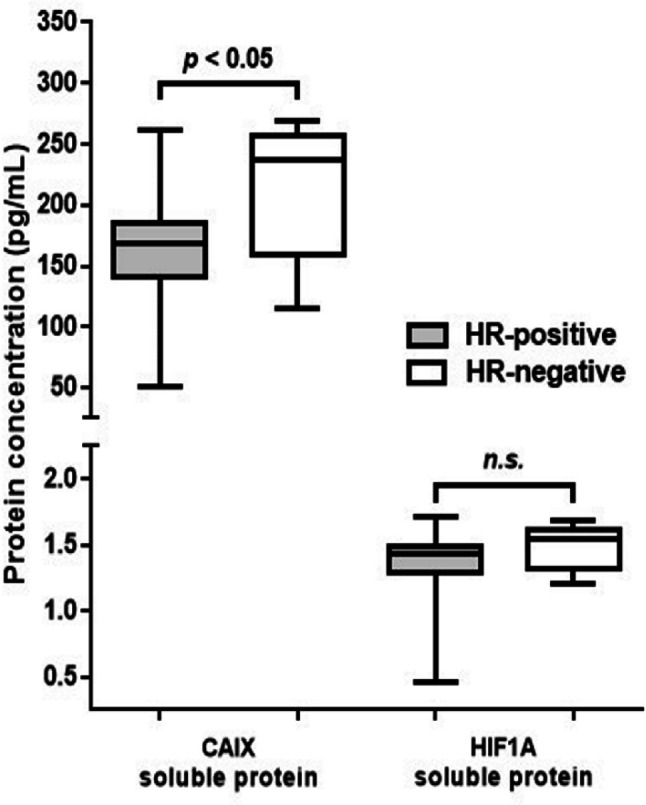
Distribution of Protein Concentration Levels of CAIX and HIF1A Based on HR

CAIX blood mRNA and CAIX soluble protein levels in HR-negative patients were higher than in HR-positive patients (p < 0.05, Table 2, Figure 1). However, the expression level of CAIX tissue mRNA, HIF1A blood mRNA, HIF1A tissue mRNA, and HIF1A soluble protein levels were similar either between the groups based on HR-presence (Figure 1, Figure 2) or molecular subtypes (Table 2). Both CAIX and HIF1A expression were having a strong correlation with histopathological grade (coefficient correlation (r) ranged from 0.7 to 0.8, p < 0.0001), but neither associated with tumor type (LABC or MBC), age, nor age group (< 40 years and ≥ 40 years) (Table 3). No associations were found between any of CAIX or HIF1A parameters with tumor types (LABC vs. MBC, data not shown). Each corresponding CAIX and HIF1A parameters are well correlated one another (r = 0.87 – 0.97, p < 0.0001) (Suppl 3).

*Survival Analysis*

Among six CAIX and HIF1A parameters, only HIF1A protein values that were not normally distributed; thus, the median was used as a cut-off (Supp. 4). Potential covariates for survival analysis are listed in Table 4. At the end of the study, among 40 patients, 23 (57.5%) had developed progression, and 12 of them had died (3 Luminal A, 2 Luminal B, 3 HER2-enriched, and 4 triple-negative). Eleven survived patients with progression were 4 Luminal A, 2 Luminal B, 3 HER2-enriched, and 2 triple-negative. 

*Univariate Hazard Ratio and Median Survival Time*

**Figure 3:**
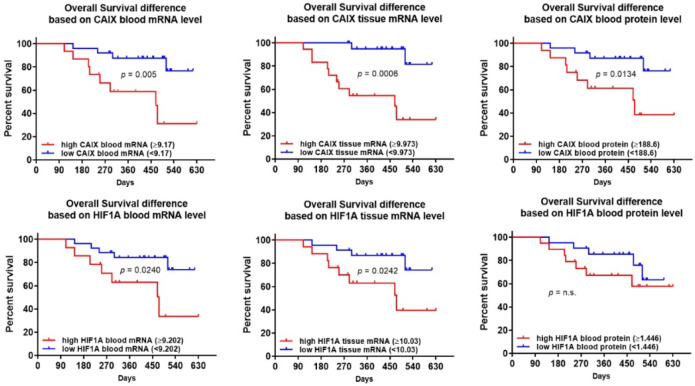
Kaplan-Meier Curves of Overall Survival between High and Low Level of CAIX and HIF1A Expression

Figure 3 showed that, individually, CAIX and HIF1A expression levels are prognostic for OS (p < 0.05), except HIF1A protein. However, none of CAIX nor HIF1A are prognostic for PFS (Supp. 5). Hazard ratio and median survival time are summarized in Table 5. 

Among six CAIX and HIF1A parameters, CAIX tissue mRNA had the highest hazard ratio for OS (8.04, 95%CI 2.45-26.39, p < 0.001). Fifty percent patients with high blood and tissue CAIX mRNA level and high blood HIF1A mRNA were survived at least 470 days, while 50% patients with high CAIX protein and tissue HIF1A mRNA were survived at least 475 days.

*Predictive Model for Survival*

Based on linear relationship availability between variables and survival outcome, for OS, only CAIX blood mRNA, CAIX tissue mRNA, CAIX soluble protein, HIF1A blood mRNA, HIF1A tissue mRNA, histopathological grade, surgical status, and tumor types were eligible for modeling. For PFS, only neoadjuvant chemotherapy type and surgical status are eligible, thus eliminating the need for further analysis.

Based on the absence of multicollinearity, covariates eligible for the model (r < 0.7) were CAIX blood mRNA, CAIX tissue mRNA, CAIX protein, HIF1A blood mRNA, HIF1A tissue mRNA, histological grade, and surgical status. Continuous data of CAIX tissue mRNA, CAIX protein, and HIF1A tissue mRNA are also eligible. One important note is that the categorical (coded) data of CAIX blood mRNA and HIF1A blood mRNA turned to be similarly coded. Hence, both variables cannot be entered into the same model (Supp. 6). 

The final model had kept CAIX tissue mRNA, surgical status, and tumor advancement as three independent predictor variables for OS (Table 6). Any of the HIF1A levels, however, did not appear as a variable with significant contribution to any model tested (p > 0.05).

**Table 1.** Patients Characteristics

Characteristics	Molecular Subtypes			
	HR-positive		HR-negative	
	Luminal A	Luminal B	HER2-enriched	Triple Negative
N (% of total 40 patients)	15 (37.5%)	6 (15%)	10 (25%)	9 (22.5%)
Age Mean (year ± SD)*	53.1		46.95	
Mean (year ± SD)	55.8 ± 15	46.3 ± 9	49.7 ± 10	45.9 ± 8
Range (year)	36 - 83	35 - 60	28 - 58	30 - 57
<40 y.o.*	3		3	
≥40 y.o.*	18		16	
Type (n)				
Locally advanced*	15		15	
Metastatic*	6		4	
Locally advanced	11	4	8	7
Metastatic	4	2	2	2
Histological grade (n)				
Grade I	1	-	-	-
Grade II	11	4	6	5
Grade III	3	2	4	4

*, Based on HR presence

**Table 2:** Mean Values of Hypoxia-Associated Biomarkers based on Molecular Subtypes

Hypoxia-associated biomarkers	Molecular Subtypes				*p*
	HR-positive		HR-negative		
	Luminal A	Luminal B	HER2-enriched	Triple Negative	
CAIX					
*mRNA*					
blood*	8.65 ± 1.3		9.75 ± 1.5		0.018
blood	8.67 ± 1.3	8.62 ± 1.3	9.73 ± 1.9	9.76 ± 1.1	0.143
tissue*	9.53 ± 1.6		10.46 ± 1.6		0.065
tissue	9.48 ± 1.5	9.64 ± 1.7	10.47 ± 1.8	10.46 ± 1.3	0.34
Sol. protein* (pg/mL)	170.2 ± 49.7		208.9 ± 52.9		0.022
*Sol. Protein*	171.3 ± 51.9	167.6 ± 48.2	204.6 ± 62.7	213.7 ± 42.8	0.154
HIF1A					
*mRNA*					
blood*	8.83 ± 1.4		9.61 ± 1.5		0.097
blood	8.78 ± 1.4	8.95 ± 1.4	9.58 ± 1.8	9.65 ± 1.1	0.432
tissue*	9.67 ± 1.6		10.43 ± 1.8		0.167
Tissue	9.65 ± 1.7	9.74 ± 1.7	10.11 ± 1.9	10.78 ± 1.7	0.458
Sol. protein* (pg/mL)	1.38 ± 0.3		1.48 ± 0.2		0.18
*Sol. protein *	1.37 ± 0.3	1.41 ± 0.2	1.46 ± 0.2	1.49 ± 0.1	0.58

*, Based on HR presence

**Table 3:** Correlation between Hypoxia-Associated Biomarkers, Age, and Histopathological Grade

Hypoxia-associated biomarkers	Correlation coefficient (r), p value	
	Age	Histopathological Grade
CAIX		
blood mRNA	-0.106, 0.514	0.765, < 0.0001
tissue mRNA	-0.187, 0.249	0.811, < 0.0001
Soluble protein	-0.102, 0.532	0.795, < 0.0001
HIF1A		
blood mRNA	-0.079, 0.626	0.738, < 0.0001
tissue mRNA	-0.138, 0.395	0.808, < 0.0001
Soluble protein	-0.027, 0.870	0.707, < 0.0001

**Table 4:** Classification of Covariates

Characteristics	Categories	n (%)
Age	<45	14 (35)
	45-54	14 (35)
	55-64	9 (22.5)
	>64	3 (7.5)
Tumor advancement	LABC	30 (75)
	MBC	10 (25)
Histopathology grade	I	1 (2.5)
	II	26 (65)
	III	13 (32.5)
Hormon receptor presence (Luminal & non-Luminal)	Positive (Luminal)	21 (52.5)
	Negative (Non-Luminal)	19 (47.5)
Neoadjuvant chemotherapy	Taxane-based	21 (52.5)
	Anthracycline-based	11 (27.5)
	Hormone-based	8 (20)
Mastectomy	No	5 (12.5)
	Yes	35 (87.5)
CAIX blood mRNA	<9.17	15 (37.5)
	≥9.17	25 (62.5)
CAIX tissue mRNA	<9.97	18 (45)
	≥9.97	22 (55)
CAIX soluble protein	<188.6 ng/mL	16 (40)
	≥188.6 ng/mL	24 (60)
HIF1A blood mRNA	<9.20	15 (37.5)
	≥9.20	25 (62.5)
HIF1A tissue mRNA	<10.03	17 (42.5)
	≥10.03	23 (57.5)
HIF1A soluble protein	<1.446 ng/mL	19 (47.5)
	≥1.446 ng/mL	21 (52.5)

**Table 5:** Log Rank test Results

	Hazard ratio	95% CI	p value	Median survival time
Overall survival				
CAIX blood mRNA	5.91	1.69-20.60	0.0053	470 days
CAIX tissue mRNA	8.04	2.45-26.39	0.0006	470 days
CAIX soluble protein	4.61	1.37-15.61	0.0134	475 days
HIF1A blood mRNA	5.91	1.69-20.60	0.0053	470 days
HIF1A tissue mRNA	3.92	1.19-12.89	0.0242	475 days
HIF1A soluble protein	1.94	0.61-6.13	n.s.	-
Progression-free survival				
CAIX blood mRNA	1.27	0.53-3.07	n.s.	-
CAIX tissue mRNA	1.12	0.48-2.63	n.s.	-
CAIX soluble protein	1.41	0.59-3.35	n.s.	-
HIF1A blood mRNA	1.27	0.52-3.07	n.s.	-
HIF1A tissue mRNA	1.12	0.38-2.09	n.s.	-
HIF1A soluble protein	1.08	0.47-2.48	n.s.	-

n.s., not significant

**Table 6:** Cox Proportional-Hazard Model for Overall Survival

Covariates	b	*p*	Exp (b)	95% CI of Exp(b)
CAIX tissue mRNA (low vs high)	1.81	0.033	60.982	1.16 - 32.13
Surgical status (no vs yes)	-1.69	0.015	0.1842	0.05 - 0.72
Tumor type (LABC vs MBC)	1.22	0.048	33.765	1.01 - 11.28

Overall Model Fit: Chi-squared = 21.872, p value, 0.0001; Note: Covariates removed from the final model: HIF1A tissue mRNA and histology grade.

## Discussion

The current prospective cohort study focused on a population of advanced breast cancer patients on their first clinical visit or referral in Makassar, South Sulawesi, the fifth largest city representative for East Indonesia region. Breast cancer incidence in Makassar ranked first (28%) among all malignancies and topped the breast cancer incidence surpassing breast cancer incidence at all other centers in Indonesia (Sarjadi and Trihartini, 2001). Most of these patients seek medical treatment at an advanced stage with local advancement or far metastasis (Ng CH, et al., 2011). Even though all patients in the metastatic stage eventually uniformly deceased to their disease, their clinical course varies greatly; some fail to survive only a few months following a recurrence, while others may survive for several years. The value of prognostic factors in early-stage breast cancer has been well established. However, reports on prognostic survival factors in treatment-naïve LABC and MBC patients are scarce, not to mention the role of hypoxia response biomarkers in these dire cases.

As similarly reported from the other region (West Indonesia), Luminal A and Luminal B are the most and the least common molecular subtype we found, respectively, and molecular subtypes are not related with age (Rahmawati et al., 2018; Setyawati et al., 2018). Molecular subtypes is an established prognostic factor for survival in the Western population (Fallahpour et al., 2017; Yang and Polley, 2019) as well as in the Asian population (Zuo et al., 2017; Abubakar et al., 2018). In MBC population, molecular subtypes (HR status), the specific site of metastasis, and metastasis-free interval were identified as independent significant prognostic factors for OS calculated from the onset of metastasis (Regierer et al., 2014). A recent report showed that the HR status (luminal vs. non-luminal categorization) has a prognostic role for survival in Indonesian breast cancer patients (n = 130) (Widodo et al., 2017). However, in that study, patients with stage IV (metastasis) are lacking. 

In the current study (clinical stages are higher than IIB /T3N0), CAIX expression was higher in HER2-enriched and triple-negative (HR-negative) patients. This finding supports the previous reports that CAIX expression was associated with the absence of hormone receptors (especially triple-negative type) and correlated with the histopathological grade (Tan et al., 2009; Pinheiro et al., 2011; Choi et al., 2013).

A study of more than 3,000 breast cancer specimens showed that a high CAIX mRNA level was significantly associated with poor survival in patients with basal-like, Luminal B, and triple-negative subtype, but not Luminal A and HER2-enriched. In triple-negative subtype only—and not in other subtypes—, a high CAIX mRNA expression is associated with shorter relapse-free survival (Ivanova et al., 2015). An older study showed that high CAIX protein level is related not only with triple-negative subtype, but also tumor size, tumor grade, chemotherapy resistance, worse OS (Tan et al., 2009), and BRCA1 mutation (Neumeister et al., 2012). An IHC-based tissue microarray study of invasive breast cancer patients (n = 276) evaluating metabolism-related proteins according to the molecular subtype, demonstrated that the expression of IGF-1, MIF, and HIF1A was correlated with the HER2-enriched subtype, while Glut-1 and CAIX expression were associated with triple-negative subtype, high histologic grade, and HR-negativity (Choi et al., 2013). 

In multivariable survival analysis, we found that a high CAIX mRNA level of tumor tissue (≥ 9.973) is an independent prognostic factor for OS, along with surgical status and tumor advancement (LABC or MBC). Unfortunately, molecular subtypes (and HR status) as a covariate in our current study did not satisfy the linearity assumptions for OS and PFS outcome as a precondition of the Cox proportional hazard model. Thus, the complementary prognostic role for OS of molecular subtypes side by side with hypoxia response biomarker in Indonesian LABC and MBC cases warrants a further study. 

We observed no correlation between the CAIX and HIF1A expression with tumor advancement (LABC or MBC). CAIX is important for hypoxic tumor cell survival by regulating acidification of the external tumor microenvironment, allowing cancer cells to adapt and further proceed invasion, then eventually develop far metastasis (Semenza, 2012). In a paired analysis between primary tumor and metastasis lesion of the same patients, CAIX protein expression (evaluated using IHC) was found significantly more frequent in distant metastases compared to their primary tumors (Jiwa et al., 2014). However, a fairly frequent conversion was also reported (42.9% negative to positive conversion, no protein expression in the primary tumor with expression in its paired metastasis). This conversion phenomenon may partly explain the absence of correlation between CAIX expression with tumor advancement (LABC or MBC) in our study.

Study regarding CAIX and HIF1A expression as biomarkers in specific LABC and MBC scenario is extremely rare, especially if using other than the IHC method (Berghuis et al., 2017). Circulating CAIX protein (evaluated using ELISA) has been reported to be useful as a biomarker for response to antiangiogenic therapy in LABC (n = 57, all HER2-negative or inflammatory cancer) but not in MBC cases (n = 23) (Brown-Glaberman et al., 2016). They reported that CAIX protein level in MBC cases is higher than in LABC cases. However, the baseline CAIX protein level in both groups is ranged widely. A recent report involving 253 MBC patients showed that elevated CAIX soluble protein level (also evaluated using ELISA) along with the presence of ≥ 5 circulating tumor cells in 7.5 ml blood predicted shorter OS and shorter PFS; however, not all involved patients were treatment-naïve (Banys-Paluchowski et al., 2018).

In the current study, HIF1A expression was not associated with either patients’ age, tumor advancement, or HR-presence; and only correlated with histopathological grade. We also did not observe any prognostic value of HIF1A expression to OS and PSF. Previous laboratory and clinical reports showed that HIF1A expression is associated with HR-negative status (Trastour et al., 2007; Wolff et al., 2017). However, our finding is somewhat contradictive to the recent meta-analysis which concludes that high HIF1A expression is an indicator of poor prognosis, even though significant heterogeneity did exist in this meta-analysis (to OS, n = 7 studies, 1,608 patients; to PFS, n = 8 studies, 1,217 patients) (Wang et al., 2014). This discrepancy may occur largely due to our limited sample number and our limited focus on treatment-naïve advanced stages only. 

HIF1A is also known to have a wide-ranging role in carcinogenesis. HIF1A expression can be detected as early as in ductal hyperplasia and atypical ductal hyperplasia (which is considered as non-malignant) as a response to a relatively hypoxic environment due to rapid cell proliferation. In subsequent stages, HIF1A plays a role to maintain this rapid proliferation rate to modulate tumor progression in later stages (Chen et al., 2010). In this study, we found that HIF1A and CAIX expression were well correlated with each other. Even though our finding is similar to a study employed tumor microarray method (TMA)(Brennan et al., 2006), it is contradictory to the other reports (Tan et al., 2009; Chen et al., 2010). This discrepancy might be explained due to the difference in experimental methods (IHC/TMA vs. RT-qPCR vs. ELISA). Previous IHC study of solid tumors showed that in perinecrotic area, CAIX is expressed without HIF1A being detected (Sobhanifar et al., 2005). It is important to note that HIF1A has a very short half-life in normoxic conditions (undetectable within minutes after re-oxygenation)(Jewell et al., 2001), such as at perinecrotic area, which is often avoided during IHC sectioning due to unpredictable immunoreactivity of epitopes in that region (True, 2008). On the other hand, CAIX protein is relatively stable and persisted much longer than HIF1A. The mRNA of CAIX is stabilized via hypoxia-activated cytoplasmic accumulation of beta-catenin, which generates CAIX transmembrane protein exhibiting high posttranslational stability with a half-life of about 40 h after reoxygenation (Pastorek and Pastorekova, 2015).

The availability of a less-subjective, quantitative tool to evaluate mRNA and soluble protein of hypoxia response should accelerate studies focusing at advanced breast cancer where patients often have limited time and therapeutic options. The conventional histopathologic approach (IHC staining or similar methods) that rely on tumor tissue sectioning remains important in histomorphologic point-of-view. However, spatial intratumoral heterogeneity is an intrinsic limitation (Aleskandarany et al., 2018). CAIX and Ki67 are among two of well-known biomarkers that are heterogeneously expressed within a particular tumor. Our colleagues reported that quantitative Ki67 obtained from the rtPCR method is similarly predictive for clinical response to neoadjuvant chemotherapy as well as its values obtained from IHC in LABC cases (Prihantono et al., 2017). Providing more sections will reduce the potential of suboptimal sampling. However, the sampling method is not the only challenge; the interpretation which is prone to substantial intra-observer and inter-observer variability is another problem (Aleskandarany et al., 2018). RT-qPCR has been considered a reasonable alternative to IHC due to several following advantages; 1) quantitative, 2) unaffected by inter-observer variability, 3) straightforward result interpretation, and 4) can be performed locally in a standardized and automated manner largely irrespective of sample size (Susini et al., 2010). However, not all amplified genes are translated into an elevated protein expression (Battle et al., 2015). Protein concentrations reflect the pathologic state of cancer cells far more directly than DNA or RNA, and proteins can be profiled effectively with several quantitative, quick, and objective methods, such as ELISA (Borrebaeck, 2017).

Among the strength of our study is the utilization of quantitative methods assessing both genomic and proteomic aspect of hypoxia-response biomarkers in a specific population of treatment-naïve LABC and MBC. However, our study is limited by the small sample numbers, which is somewhat complicated for multivariate modeling. Another limitation of this study is the absence of a comprehensive (whole body) diagnostic approach to establish LABC and MBC, such as whole-body bone scintigraphy or PET/CT scan. Such a comprehensive diagnostic approach is critical in developing countries like ours, where breast cancer patients mostly visit the medical centers at their late advanced stage. A large-scale multicenter study with holistic diagnostic tools for LABC and MBC patients may provide a more robust prognostic cut-off values of these tumor hypoxia biomarkers.

As a conclusion , the current study demonstrated the prognostic role of tumor tissue mRNA of CAIX for OS in the patient subpopulation of treatment-naïve advanced breast cancer. This finding may help clinician to refine the treatment plan including therapeutic options of LABC and MBC patients. Clinical translation of the current finding, including the prognostic role of HIF1A, warrants a future study.
